# Bone-Felon Arrested by Congelation

**Published:** 1872-06

**Authors:** 


### Bone-Felon Arrested by Congelation.

James B. Walker, M. D., of St. Louis County, Missouri, writes as follows to the *Medical Archives:*

Not long since I was consulted by a young lady, who was suffering from an incipient felon. The distinguishing characteristics of the painful affection were already manifest—pain, throbbing, some tumefaction, and the nervous excitement, indicated plainly what was in advance, unless the inflammation was arrested, and the command was, arrest it at all hazards. The starting point had been two days previous to her application for treatment. I could think of nothing offering such a prospect of success as cold, as low as the freezing point Adding equal parts of snow and salt in a tumbler, I placed the finger, it being the middle one, in the freezing mixture. For a few seconds there was an increase of the sensibility of the part, and it was with difficulty I could persuade her to hold the finger in the mixture. By degrees the pain subsided, and at the end of two minutes perfect insensibility had followed. I removed the finger, and after a few minutes the sensibility returned, and with it came the pain throbbing, etc. The application was renewed, the pain again ceased, and insensibility ensued. This was repeated as often as the pain returned, and in about two hours, alternating the application and removal there was no return of the painful sensations, and the difficuty entirely ceased, and there was no felon. The induration remained several days, and the skin gradually exfoliated. The young lady was highly gratified at the success of the treatment.

